# Ectopic expression of *GhSAMDC*_*1*_ improved plant vegetative growth and early flowering through conversion of spermidine to spermine in tobacco

**DOI:** 10.1038/s41598-020-71405-z

**Published:** 2020-09-02

**Authors:** Huaguo Zhu, Wengang Tian, Xuefeng Zhu, Xinxin Tang, Lan Wu, Xiaoming Hu, Shuangxia Jin

**Affiliations:** 1 College of Biology and Agricultural Resources, Huanggang Normal University, Huanggang, 438000 Hubei China; 2 Hubei Key Laboratory of Economic Forest Germplasm Improvement and Resources Comprehensive Utilization, 438000, Huanggang, Hubei China; 3 College of Agronomy, Shihezi University, Shihezi, 832000 Xinjiang China; 4 College of Plant Science and Technology, Huazhong Agricultural University, Wuhan, 430070 Hubei China

**Keywords:** Plant physiology, Developmental biology

## Abstract

Polyamines play essential roles in plant development and various stress responses. In this study, one of the cotton S-adenosylmethionine decarboxylase (SAMDC) genes, *GhSAMDC*_*1*_, was constructed in the pGWB17 vector and overexpressed in tobacco. Leaf area and plant height increased 25.9–36.6% and 15.0–27.0%, respectively, compared to the wild type, and flowering time was advanced by 5 days in transgenic tobacco lines. Polyamine and gene expression analyses demonstrated that a decrease in spermidine and an increase in total polyamines and spermine might be regulated by *NtSPDS*_*4*_ and *NtSPMS* in transgenic plants. Furthermore, exogenous spermidine, spermine and spermidine synthesis inhibitor dicyclohexylamine were used for complementary tests, which resulted in small leaves and dwarf plants, big leaves and early flowering, and big leaves and dwarf plants, respectively. These results indicate that spermidine and spermine are mainly involved in the vegetative growth and early flowering stages, respectively. Expression analysis of flowering-related genes suggested that *NtSOC*_*1*_, *NtAP*_*1*_, *NtNFL*_*1*_ and *NtFT*_*4*_ were upregulated in transgenic plants. In conclusion, ectopic *GhSAMDC*_*1*_ is involved in the conversion of spermidine to spermine, resulting in rapid vegetative growth and early flowering in tobacco, which could be applied to genetically improve plants.

## Introduction

Polyamines, including putrescine (diamine), spermidine (triamine), and spermine (tetraamine), play various roles in plants^1,2^. Many reports indicate that polyamines are affect the fluidity of the lipid membrane and participate in the biotic and abiotic stress responses^3–9^. Furthermore, polyamines have been reported to be involved in various physiological processes^9–14^.

The polyamine metabolic pathways have been elucidated in plants^15^. Putrescine originates from ornithine or arginine, catalysed by ornithine decarboxylase, or arginine decarboxylase, agmatine iminohydrolase and N-carbamoyl putrescine amidohydrolase. Spermidine and spermine are derived from putrescine and are catalysed by spermidine and spermine synthases, respectively. S-adenosylmethionine decarboxylases (SAMDCs) catalyse the S-adenosylmethionine decarboxylation reaction and provide an aminopropyl group, which is involved in spermidine and spermine synthesis. *SAMDC* transcripts have been analysed in a wide variety of plant species, often with higher transcript levels detected in reproductive than vegetative organs^16–21^. Further evidence that polyamines are required for growth and development comes from analysing the *Arabidopsis thalian* bud2 mutant. This mutant has an inactivated *AtSAMDC*_*4*_ gene and an enlarged vascular phenotype^22^. Moreover, *AtSAMDC*_*1*_ is involved in an interaction between beet severe curly top virus and *Arabidopsis* plants^23^. Numerous studies have reported upregulation of *SAMDC* in response to various stressors, including drought, salt, high or low temperature and oxidative stress^24–28^. Changes in polyamine transporters reveal a significant role of spermidine in the timing of flowering^12^.

Flowering is a critical reproductive function that is supervised by a complex interaction between environmental and internal factors. Plants have evolved coordinating flowering pathways, including gibberellin, vernalisation, photoperiod and ageing pathways^29^. These pathways converge a series of downstream integrators, including flowering locus T (FT), apetala1 (AP_1_), suppressor of over-expression of CO1 (SOC_1_), and LEAFY (LFY)^30^. Expression analyses of these genes have been used to evaluate flowering. Many studies have shown that exogenous polyamines and polyamine synthesis inhibitors affect flower bud differentiation and development^12,31,32^.

Although polyamines have been implicated in controlling developmental processes, their fundamental roles remain unknown. In this study, ectopic expression of *GhSAMDC*_*1*_ resulted in a prompt decrease in spermidine and an increase in spermine in transgenic plants. In addition, rapid vegetative growth and early flowering occurred in transgenic plants. Complementary tests further verified the rapid changes in spermidine and spermine involved in early flowering. Taken together, these results indicated that polyamine homeostasis is involved in plant flowering development, and that some polyamine-related genes might be applicable for genetic improvement of crops.

## Results

### Identification of transgenic tobacco plants

**Figure 1:**
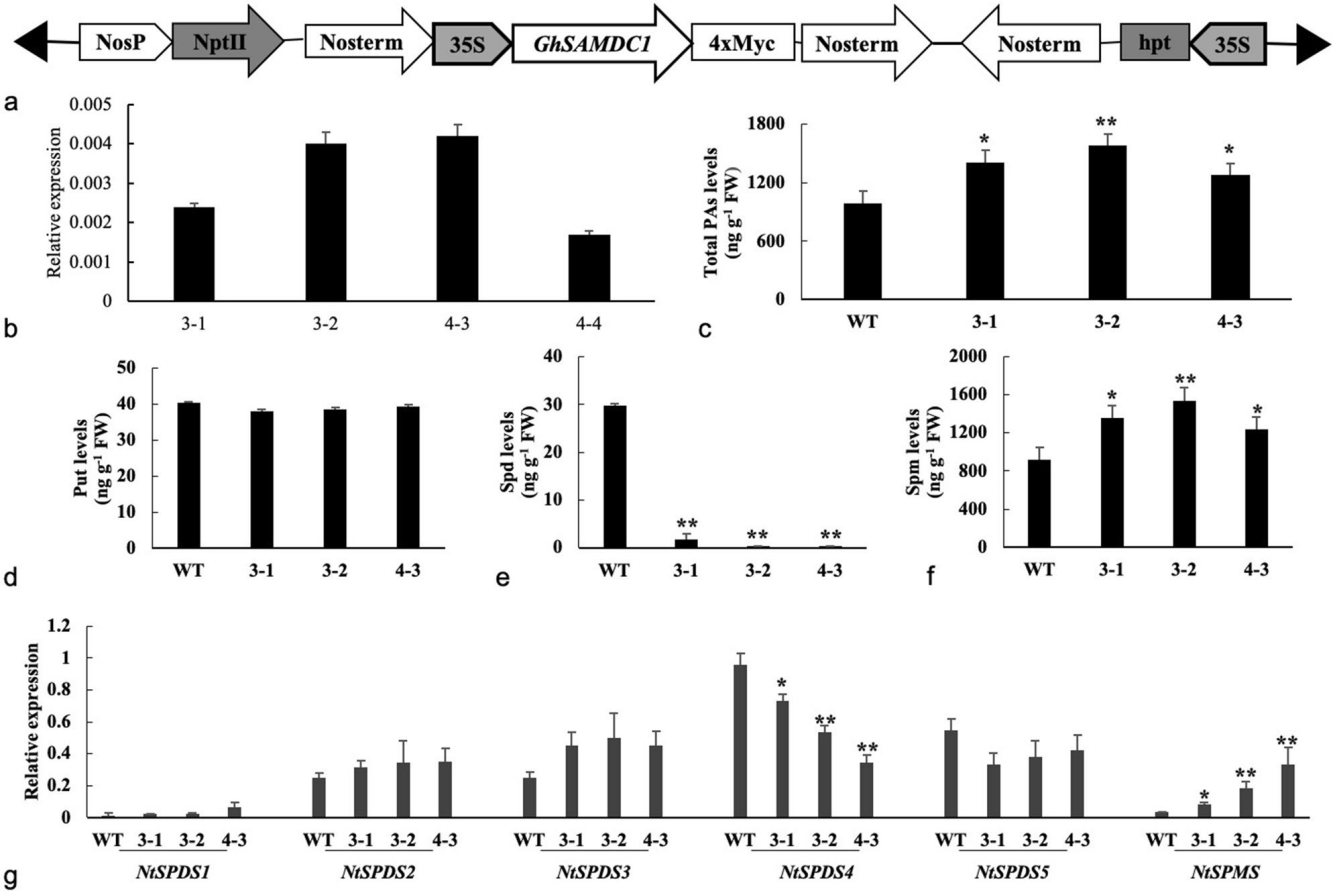
Transgenic tobacco lines expressing *GhSAMDC*_*1*_ (WT, 3–1, 3–2, 4–3 and 4–4 means wild type and transgenic lines 3–1, 3–2, 4–3 and 4–4. Leaves from three 30 days old plants were sampled for polyamine detection and expression analysis. Data are means of 3 replicates ± SE. Bars labeled with * and ** are significantly different at P < 0.05 and 0.01, respectively. WT as control for significance analysis). (**a**) Expression vector of *GhSAMDC*_*1*_; (**b**) q-RT PCR analysis of *GhSAMDC*_*1*_; (**c**–**f**) total polyamines, putrescine, spermidine, spermine levels; (**g**) q-RT PCR analysis of *NtSPDS*_*1- 5*_ and *NtSPMS.*

The *SAMDC* gene from cotton (*GhSAMDC*_*1*_) driven by the 35S promoter was transformed into tobacco to investigate the effect in vivo (Fig. 1a). Three transgenic lines with different transcription levels were selected for further analysis (Fig. 1b). As is shown in Fig. 1c–f, increases in total polyamines and spermine were observed in the transgenic lines; however, the opposite results were found for spermidine. Furthermore, transcription of *NtSPDS*_*1-5*_ and *NtSPMS* was detected, but only the expression levels of *NtSPDS*_*4*_ and *NtSPMS* were notably different between the wild-type and transgenic lines.

### Ectopic expression of *GhSAMDC*_*1*_ improves vegetative growth and early flowering in transgenic tobacco plants

**Figure 2:**
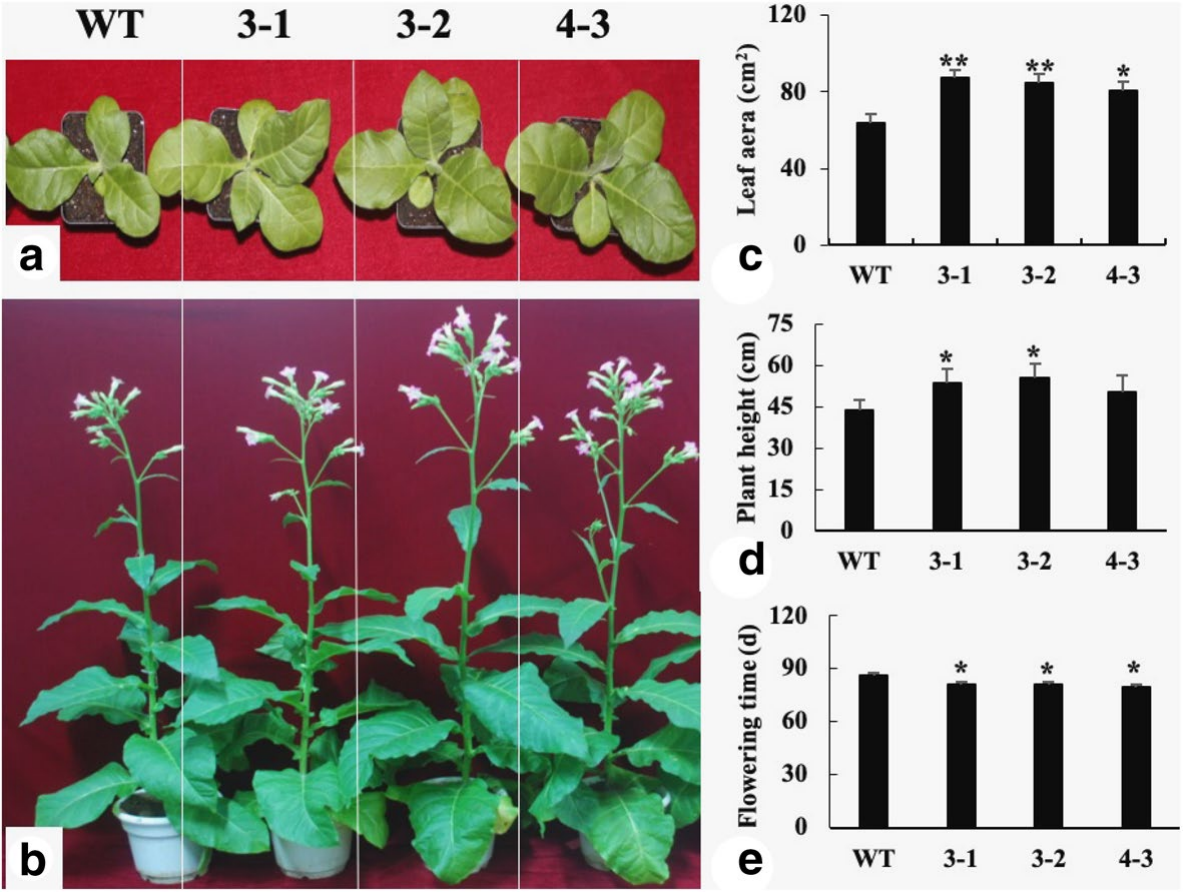
Morphological phenotypes of wild type and transgenic plants (WT, 3–1, 3–2 and 4–3 means wild type and transgenic lines 3–1, 3–2 and 4–3. Data are means of 3 replicates ± SE. Bars labeled with * and ** are significantly different at P < 0.05 and 0.01, respectively. WT as control for significance analysis).

Wild-type and transgenic plants were planted in a climate-controlled greenhouse (16-h light and 8-h dark cycle at 25 °C) and their performance was observed and recorded (Fig. 2a,b). Transgenic plants showed rapid vegetative growth accompanied by larger leaf area and greater plant height compared to wild-type plants (Fig. 2c,d). Furthermore, the flowering time was advanced by 5 days in the transgenic lines (Fig. 2e). Overall, ectopic expression of *GhSAMDC*_*1*_ improved vegetative growth and early flowering in transgenic tobacco.

### Efficient conversion of spermidine to spermine promotes flowering in transgenic plants

**Figure 3:**
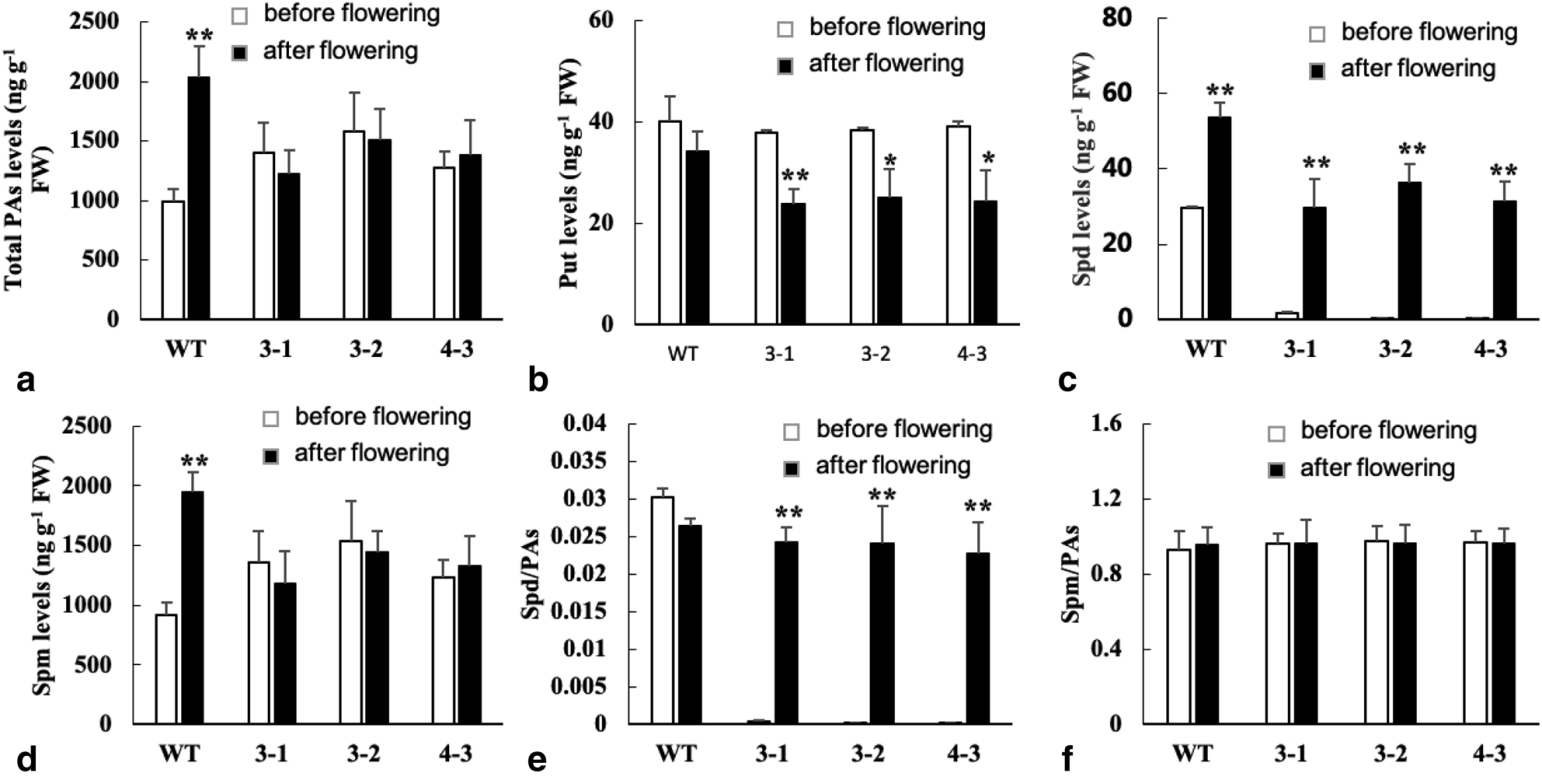
Polyamine levels in wild type and transgenic line plants (WT, 3–1, 3–2 and 4–3 means wild type and transgenic lines 3–1, 3–2 and 4–3. Before flowering and after flowering leaves sampled from 30 and 90 days old plants. Data are means of 3 replicates ± SE. Bars labeled with * and ** are significantly different at P < 0.05 and 0.01, respectively. Before flowering as control for significance analysis).

Detecting polyamines in wild-type and transgenic plants suggested that total polyamines, particularly spermidine content, changed significantly before and after flowering. Total polyamine content in the wild type was lower before flowering (Fig. 3a). Putrescine concentration decreased after flowering in transgenic plants (Fig. 3b). In contrast, spermidine concentration increased after flowering, particularly in transgenic plants (Fig. 3c). Furthermore, spermine concentration increased and remained stable before and after flowering in wild-type and transgenic plants, respectively (Fig. 3d), which resulted in a dramatic increase in the ratio of spermidine/polyamine in transgenic and a stable ratio in the wild type plants (Fig. 3e). The ratio of spermine/polyamine remained stable before and after flowering (Fig. 3f). These results indicate that a lower concentration of spermidine and a rapid conversion of spermidine to spermine before flowering could result in an early flowering phenotype in transgenic plants.

### Exogenous spermidine and its inhibitor DCHA alter the vegetative growth of tobacco

**Figure 4:**
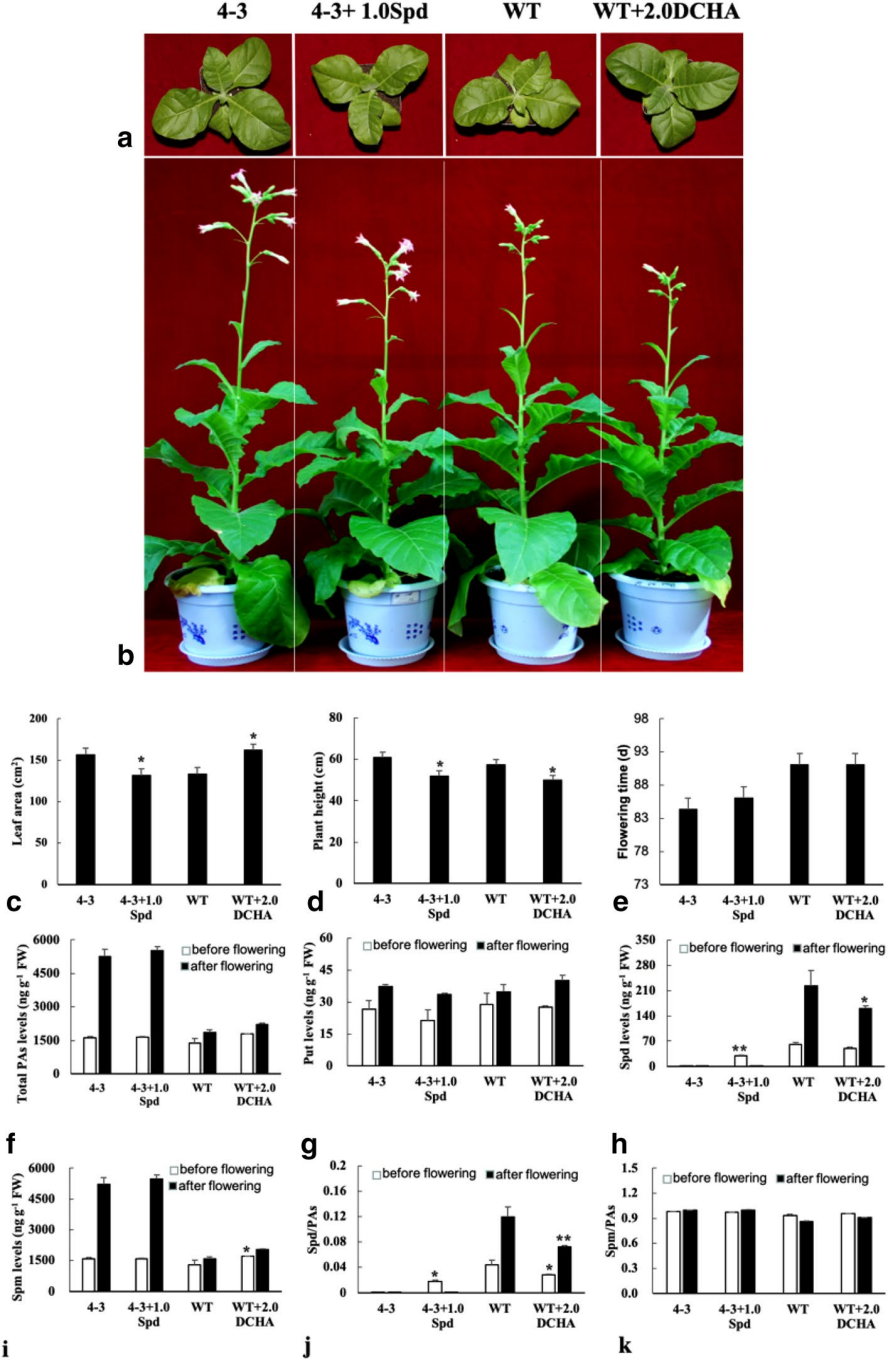
Spermidine involved in vegetative growth and the decrease of the ratio of spermidine/ polyamines stimulates early flowering in tobacco (WT and 4–3 means wild type and transgenic line 4–3. 30 and 60 days old plants were used for measure of leaf area and plant height, respectively. Data are means of 3 replicates ± SE. 4–3 and WT as 4–3 + 1.0 Spd and WT + 2.0 DCHA controls for significance analysis, respectively). (**a**–**e**) 1.0 mM spermidine and 2.0 mM DCHA inhibited vegetative growth; (**f**–**k**) polyamines changes of wild type and transgenic line 4–3 treated with 1.0 mM spermidine and 2.0 mM DCHA before and after flowering).

To elucidate the effect of spermidine on flowering in tobacco, 1.0 mM spermidine and 2.0 mM DCHA, a spermidine biosynthetic inhibitor, were applied to transgenic line 4–3 and wild-type plants, respectively. The leaf area of the transgenic line 4–3 plants treated with spermidine decreased compared with the control, and DHCA treatment increased leaf area in wild-type treated plants (Fig. 4a,c). Exogenous spermidine and DCHA hindered transgenic and wild-type plant growth (Fig. 4b,d). Spermidine and DCHA did not have a distinct effect on the timing of tobacco flowering (Fig. 4b,e). Before flowering, exogenous spermidine increased spermidine content in transgenic line 4–3, and exogenous DCHA increased spermine content in the wild type (Fig. 4h,i). After flowering, only exogenous DCHA decreased spermidine in the wild type (Fig. 4h). Furthermore, apart from the putrescine and spermidine content in transgenic lines 4–3 and 4–3 treated with spermidine, spermine and total polyamine contents (including putrescine, spermidine and spermine) were highly elevated (∼2.0 fold, P < 0.05) compared to that before flowering, and spermidine contents remarkably increased (∼2.0 fold, P < 0.05) in wild-type and wild-type treated DCHA after flowering (Fig. 4f–i). The ratios of spermidine/polyamines increased in transgenic lines 4–3 treated with spermidine and decreased in wild-type treated DCHA (Fig. 4j). The ratio of spermine/polyamines remained unchanged (Fig. 4k). These results suggest that spermidine might be involved in vegetative growth.

### Exogenous spermine accelerates vegetative growth and promotes early flowering in wild-type tobacco

**Figure 5:**
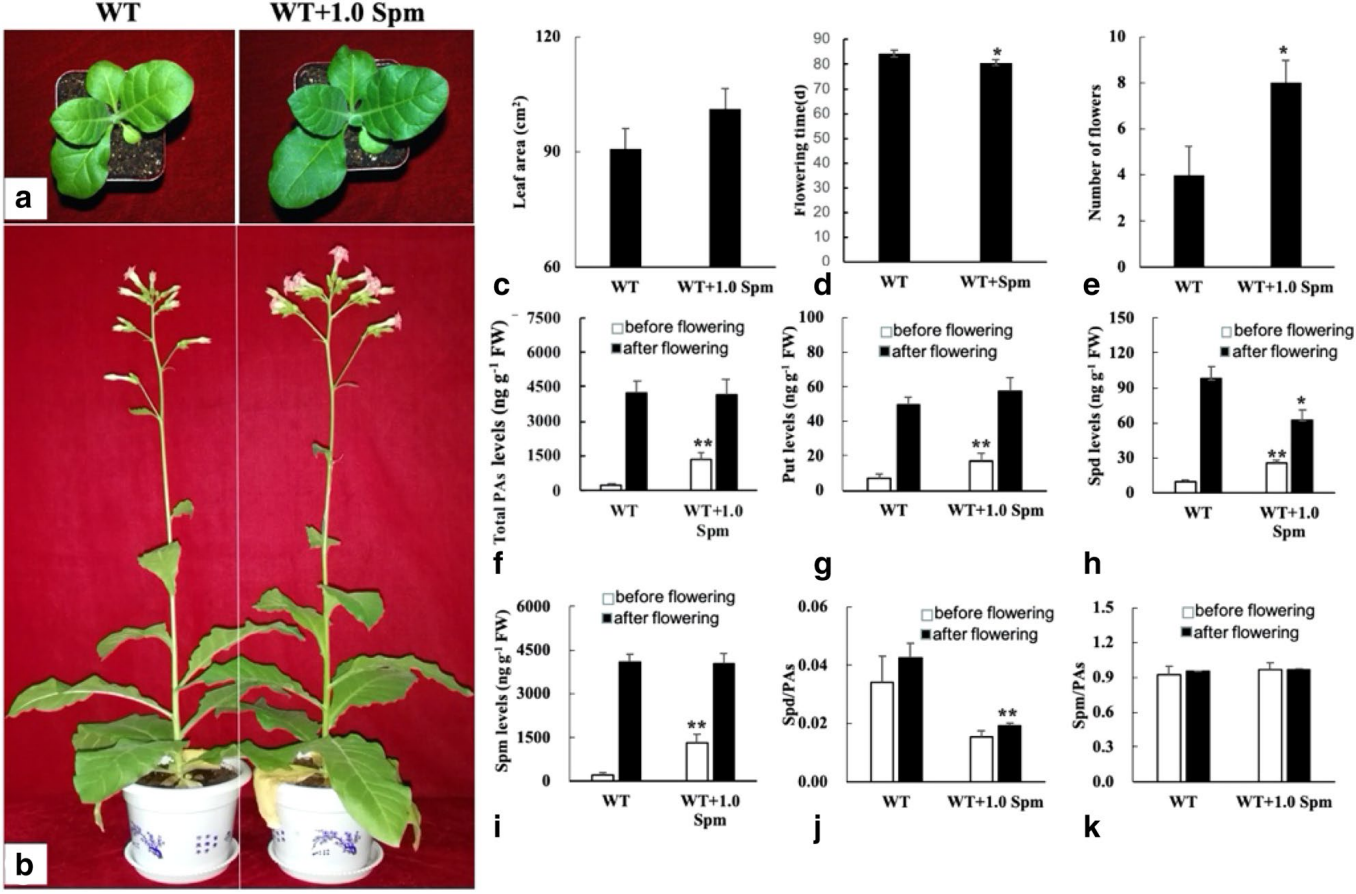
Effects of spermine on growth, flowering and polyamines (20 and 90 days old plants were used for measure of leaf area and flowers number, respectively. Data are means of 3 replicates ± SE. Bars labeled with * and ** are significantly different at P < 0.05 and 0.01, respectively. WT as control for significance analysis). (**a**–**e**) 1.0 mM exogenous spermine promoted vegetative growth and promoted flowering in wild type plants; (**f**–**k**) 1.0 mM exogenous spermine promoted polyamines concentration and lowered the ratio of spermidine/polyamines before flowering, and lowered spermidine concentration and the ratio of spermidine/polyamine after flowering in wild type plants.

To clarify the effect of spermine on tobacco vegetative growth and flowering, 1.0 mM exogenous spermine was applied to wild-type plants. Exogenous spermine accelerated vegetative growth and promoted early flowering in wild-type tobacco. Moreover, the phenotype of the wild-type plants treated with exogenous spermine was similar to plants ectopically expressing *GhSAMDC*_*1*_ (Fig. 5a–e). Increased polyamine content (including total polyamines, putrescine, spermidine and spermine) was evident after exogenous spermine treatment before flowering. Also, the contents of polyamines were enhanced after flowering in the wild-type and spermine-treated plants (Fig. 5f–i). Only spermidine concentration and the ratio of spermidine/polyamines decreased in spermine-treated plants after flowering (Fig. 5j,k), which further suggested that the decrease in spermidine might be a typical in flowering tobacco.

### Flowering-related genes activate in early flowering transgenic tobacco plants

**Figure 6:**
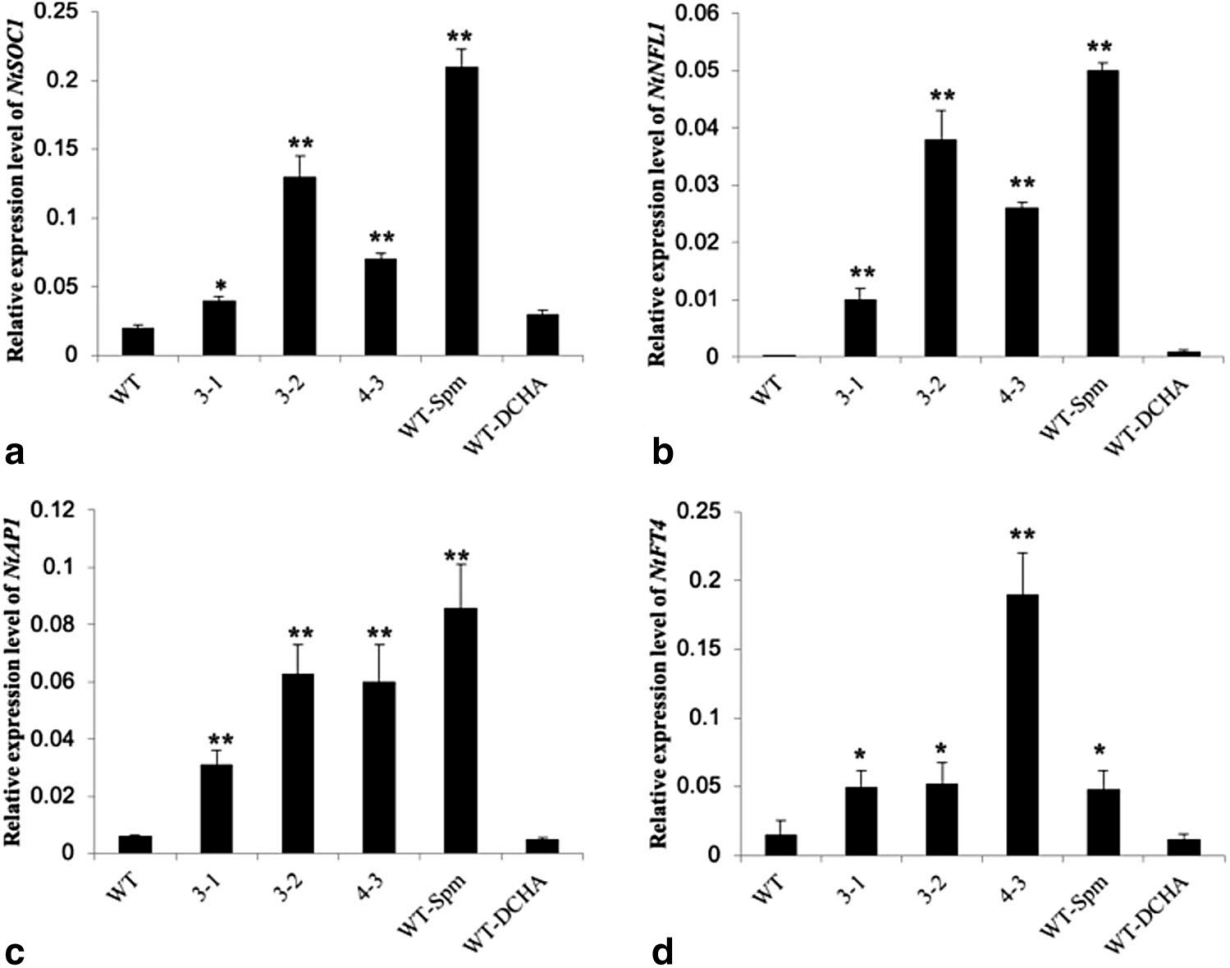
Analysis of genes related to flowering by qRT PCR (WT, 3–1, 3–2, 4–3, WT-Spm and WT-DCHA means wild type, transgenic line 3–1, 3–2, 4–3, wild type treated with 1.0 mM Spm and wild type treated with 2.0 mM DCHA. Leaves from 30 days old plants were sampled for gene expression analysis, three biological replicates. Data are means of 3 replicates ± SE. Bars labeled with * and ** are significantly different at P < 0.05 and 0.01, respectively. WT as control for significance analysis).

Transcription analysis of flowering-related genes indicated that *NtSOC*_*1*_, *NtNFL*_*1*_, *NtAP*_*1*_ and *NtFT*_*4*_ were upregulated in transgenic and spermine-treated lines, suggesting that these genes might be linked to the decrease in spermidine or the increase in spermine (Fig. 6). The relationship between the abundance of polyamines and the transcription levels of flowering-related genes requires more research.

### Polyamines are spatially distributed in wild and transgenic tobacco

**Figure 7:**
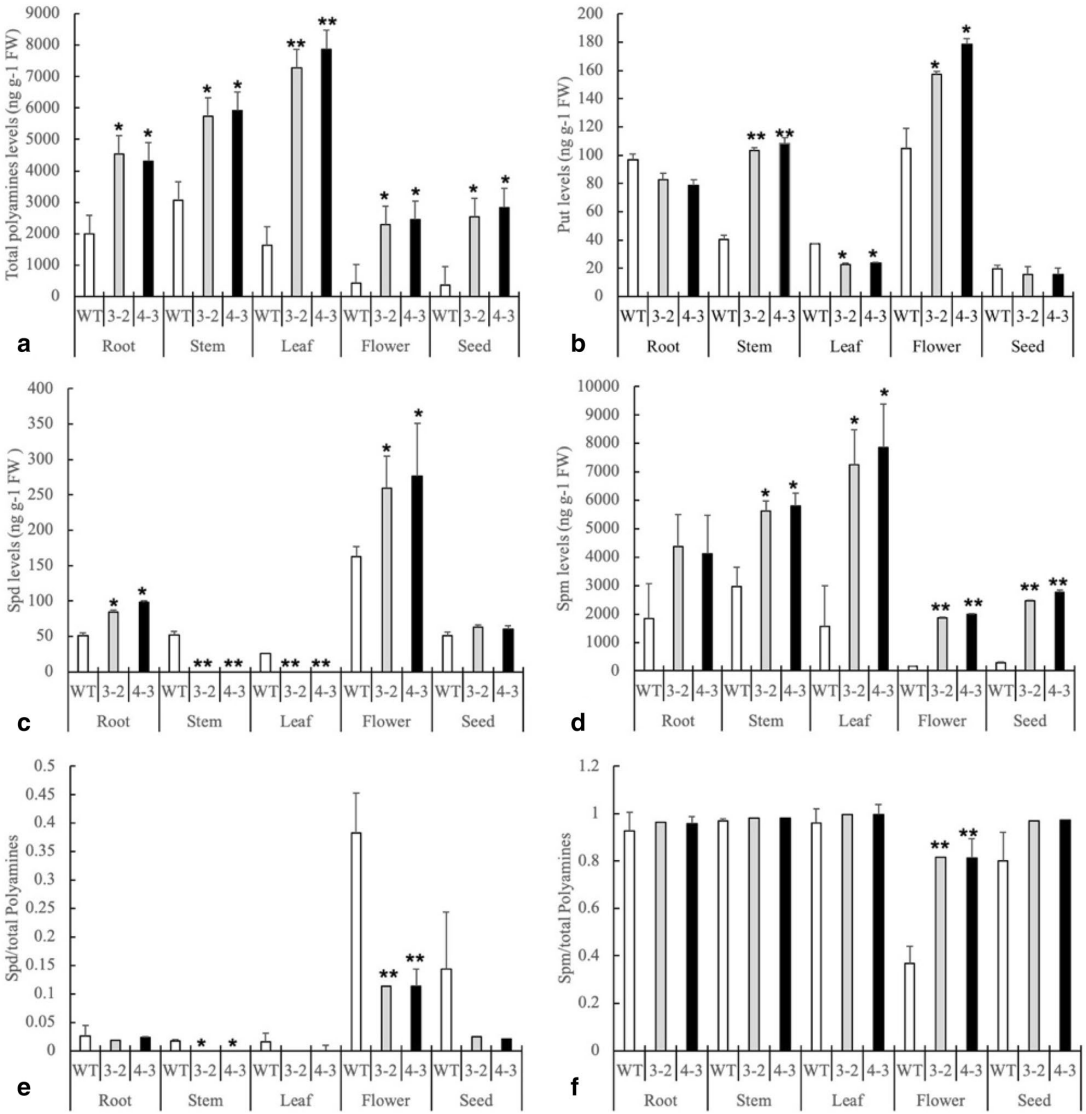
Polyamine levels in various organs (WT, 3–2 and 4–3 means wild type and transgenic line 3–2 and 4–3. Root, stem and leaf sampled from 30 days old plants, flower sampled from 90 days old plants at same position, and seed sampled from mature seed, three replicates were repeated each time. Data are means of 3 replicates ± SE. Bars labeled with * and ** are significantly different at P < 0.05 and 0.01, respectively. WT as control for significance analysis).

Detecting polyamines in diverse tissues demonstrated that the polyamines were spatially distributed in different tissues during plant development. Notably, ectopic expression of *GhSAMDC*_*1*_ resulted in increases of total polyamines in all tissues (Fig. 7a). Various changes in putrescine, spermidine and spermine occurred in different tissues between the wild-type and transgenic lines. Putrescine content decreased in leaves, whereas it increased in stems and flowers (Fig. 7b). Furthermore, spermidine concentration increased in roots and flowers, and decreased in stems and leaves compared to the wild-type (Fig. 7c); however, spermine increased in all tissues except for roots (Fig. 7d). Spermidine increased and the ratio of spermidine/polyamines decreased in flowers of the transgenic lines compared with other tissues (Fig. 7c,e), which was in line with the rapid change in spermidine content before and after flowering (Fig. 3c). This result implies that spermidine might play a key role in flower bud differentiation. Finally, only the ratio of spermine/total polyamines increased in flowers in the transgenic lines compared to the wild type, indicating that spermine might be involved in flower development in tobacco (Fig. 7f).

### Spermidine and spermine affect flower development in tobacco

**Figure 8:**
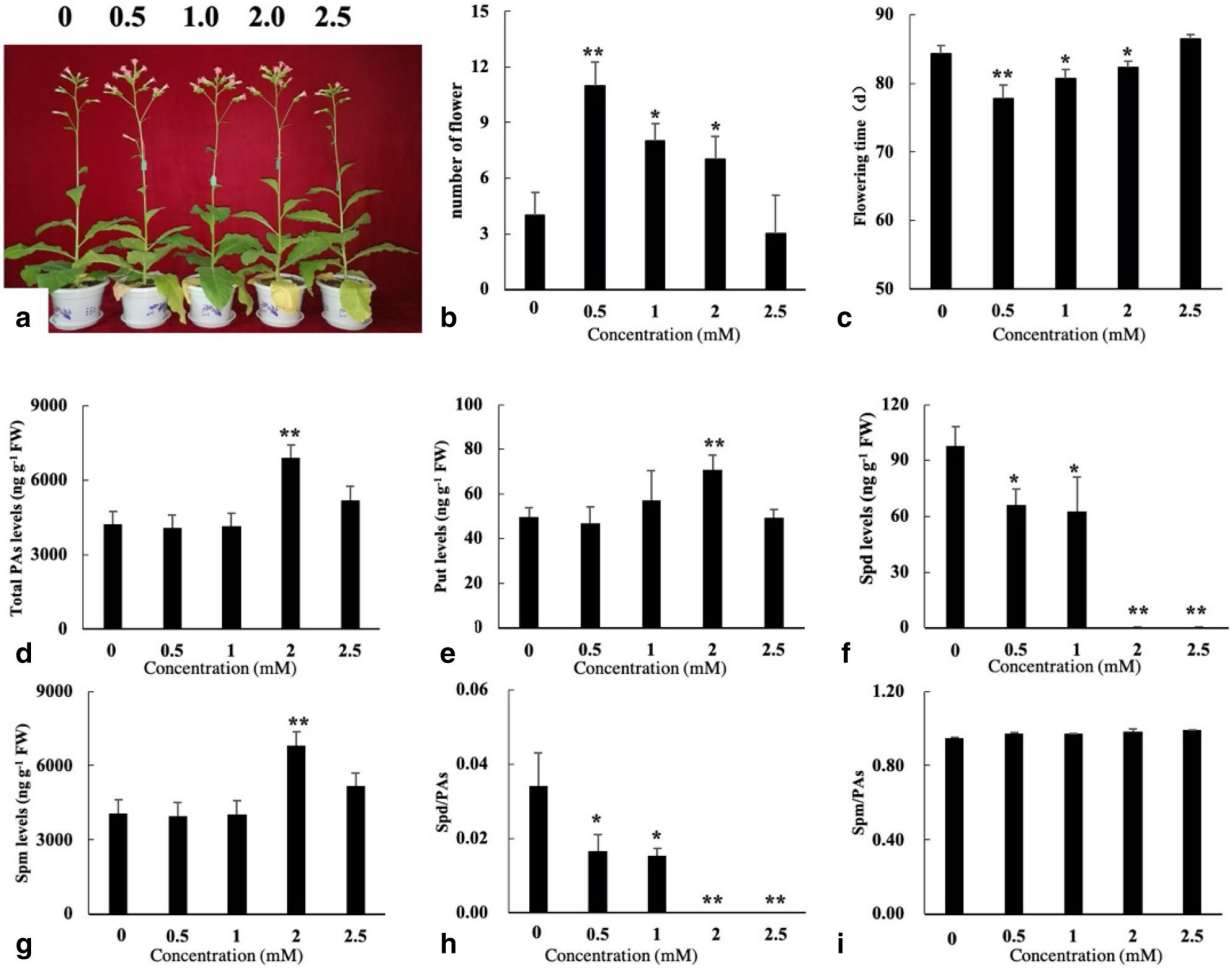
Growth, flowering, and polyamine levels as a function of concentration for spermine treatment (0, 0.5, 1, 2 and 2.5 means 0 mM, 0.5 mM, 1 mM, 2 mM and 2.5 mM exogenous spermine for different treatments. 90 days old plants were used for flower number count. Data are means of 3 replicates ± SE. Bars labeled with * and ** are significantly different at P < 0.05 and 0.01, respectively. 0 mM treatment as control for significance analysis). (**a–c**) phenotype of flowering in different exogenous spermine treatments; (**d–i**) putrescine, spermidine, spermine, total polyamines concentration, and the ratio of spermidine/polyamines and spermine/polyamines in different spermine concentration treatments, respectively.

To further elucidate the effect of spermine on flowering in tobacco, various concentrations of spermine were sprayed onto the leaves of wild-type tobacco plants. The results showed that 0.5 mM exogenous spermine was the most effective for regulating flowering time, and flowering time was extended as the exogenous spermine concentration was increased (Fig. 8a–c). The polyamines detection analysis suggested that the highest content (total polyamines, putrescine and spermine) occurred under the 2.0 mM exogenous spermine treatment (Fig. 8d,e,g). Moreover, spermidine content and the ratio of spermidine/total polyamines decreased following the increase in exogenous spermine (Fig. 8f,h), whereas the ratio of spermine/total polyamines remained the same (Fig. 8i). These results indicate that exogenous spermine affects the content of endogenous polyamines, and together with endogenous spermidine affects the development of tobacco flowers.

## Discussion

Studies have shown that polyamines exhibit tissue- and organ-specific distribution patterns in plants^14,33^. The most abundant polyamine in leaves is putrescine, and its level is three times higher than that of spermidine and spermine, whereas spermidine is the most abundant in other organs^33^. Different types of polyamines show different localisation patterns within cells. For instance, putrescine accumulates in the cytoplasm, and spermine accumulates in the carrot cell wall^34^. The distribution patterns of polyamines may be related to their unique functions. In general, more vigorous plant growth and metabolism is associated with greater polyamine biosynthesis and higher polyamine content^35^. Spermidine synthesis related-genes, such as the *SAMDC* and *SPDS* genes, are highly expressed in flower tissues, and *SAMDC* RNAi plants develop sterile pollen^16,17^. In this study, along with the increased vegetative growth and earlier flowering in the transgenic lines, ectopic expression of *GhSAMDC*_*1*_ increased total polyamines and spermine, and reduced spermidine. Expression analysis of *NtSPDS*_*1-5*_ and *NtSPMS* demonstrated that *NtSPDS*_*4*_ and *NtSPMS* were downregulated and upregulated, respectively, in the transgenic lines (Fig. 1), indicating that the conversion of spermidine to spermine might be regulated by *NtSPDS*_*4*_ and *NtSPMS* in tobacco. These data indicate that spermidine is temporally and spatially distributed in different tissues and primarily plays a vital role in cell differentiation and flower development.

In plants, homeostasis is performed by modulation of PA biosynthesis, conjugation, catabolism, and transport, and polyamines are involved in many complex stress and developmental processes throughout the plant lifespan^10^. Xu et al. (2015) reported that the exogenous polyamine could accelerates chrysanthemum bud differentiation, while a high content of polyamines in apical meristems is conducive to initiate and maintain differentiation of chrysanthemum buds. *A. thaliana* flowers contain more polyamines than any other organs, and adding exogenous polyamines to flowering plants significantly promotes their flowering response^36^. Moreover, an earlier peak in polyamine content in tissues resulted in an earlier maturing cultivar^37^. Similar results were found in Dendrobium nobile, in which the higher putrescine and spermidine levels in leaves resulted in more flower buds and flowers, and larger average flower diameters^38^.

Many studies have reported that polyamines, particularly spermidine and spermine, are involved in the initiation and development of flowering, but their effect on flowering is unclear. Applying a polyamine synthase inhibitor in the growth medium reduces spermidine content in *Arabidopsis*, almost completely inhibiting bolting and flowering, and the resumption of bolting and flowering is achieved after the plant is transferred to inhibitor-free medium^31,36,39^. However, injecting *Arabidopsis* roots with spermidine results in delayed flowering under indulgent flowering conditions^36^. In addition, a delay of flowering time is associated with a significant increase in the spermidine level in leaves before flowering^12^, while the spermidine concentration increases sharply at the initiating stage of primary flowers^40^. These results suggest that spermidine may be a physiological determinant of early flowering^41^.

In contrast, another study showed that applying spermine improves the quality of cut roses and extends their vase life by 3 days^32^. A low content of polyamines (mainly putrescine and spermidine) in rapeseed is conducive to initiate flower bud differentiation, while increased polyamine content is conducive to flower bud development^37^. In this study, transgenic tobacco plants and plants treated with exogenous spermine blossomed significantly earlier. Spermidine content in the leaves of transgenic plants decreased significantly before flowering, while spermidine content and the expression levels of flowering-related genes were upregulated, indicating that spermidine and spermine may play crucial roles in flower bud differentiation and flower development, respectively.

Overall, rapid vegetative growth and early flowering were demonstrated in overexpressed *GhSAMDC*_*1*_ tobacco plants. The polyamine detection and gene expression analyses showed that increases in total polyamines and spermine might be regulated by *NtSPDS*_*4*_ and *NtSPMS* in transgenic plants. Complementary tests suggested that spermidine and spermine are mainly involved in vegetative growth and early flowering, respectively. Consistent with the early flowering phenomenon, upregulation of flowering-related genes occurred in the transgenic plants. Thus, ectopic *GhSAMDC*_*1*_ was involved in the conversion of spermidine to spermine, which resulted in rapid vegetative growth and early flowering in transgenic tobacco; these findings suggest a suitable candidate gene to regulate plant growth and flowering in the future.

## Methods

### Plant materials and treatments

Wild-type and homozygous T_3_ transgenic tobacco seeds (including 3–1, 3–2 and 4–3) were surface-sterilised with 2% NaClO and washed five times with sterile water. The sterile seeds were then suspended in 0.2% agar and plated on 1⁄2 Murashige and Skoog (MS) medium plus 1.5% sucrose^42^. Seedlings were selected and planted in a climate-controlled greenhouse (16 h light and 8 h dark cycle at 25 °C), and leaves from the same position on 30-day-old and 90-day-old plants were sampled to detect polyamine and perform a gene expression analysis. For the complementary tests, 1 mM spermidine, 1 mM spermine and 2 mM dicyclohexylamine (DCHA) were sprayed on 60-day-old plant leaves once per day until the emergence of a floral phenotype. Each experiment was repeated twice, and three technical replicates were repeated each time.

### RNA isolation and quantitative reverse transcriptase-polymerase chain reaction (qRT-PCR) analysis

Total RNA was extracted from the samples using the modified CTAB method^43^. A total of 2 μg of RNA was treated with DNase I and used for the first-strand cDNA synthesis with MLV reverse transcriptase (Takara, Shiga, Japan), according to the manufacturer’s instructions. qRT-PCR was performed using Power SYBR Green Master (Roche, Basel, Switzerland) on a Roche Light Cycler 480 system (Roche), as described previously^44^. The reaction was run as follows: pre-incubation at 94 °C for 2 min, 40 cycles of 94 °C for 20 s, 58 °C for 20 s and 72 °C for 20 s. The actin gene (*Tac9*; X69885) was used as the reference gene, and the relative 2^−∆ct^ quantification method was used to evaluate quantitative variation^45^. Three biological replicates and three technical repeats were run. The qRT-PCR primers are listed in Table 1.

**Table 1:** Primers used for qRT-PCR in this study.

Gene name	Sense primer sequence (5′-3′)	Antisense primer sequence(5′-3′)
*NtAP1* (LOC107761268)	TTCAAAGAGGAGGGGAGGTT	CAAGCGTCTCTCTGCGTATG
*NtNFL1 *(LOC107822738)	GGAGCGAGGAGAGAATGTTG	AGCGTTGCTTCGTTCAGAAT
*NtSOC1 *(LOC107818776)	AAACGCAGAAATGGTTTGCT	TTCCCCAACTTGGTTTTCAG
*NtSPDS1* (LOC107763093)	AGGAGTGTGCAGCATTTGTG	CCAGCCAGGAAGAACAGAAG
*NtSPDS2* (LOC107828197)	AGGTGTCGCGTCATTCTTCT	CGAACAACTCTTGTGCTGGA
*NtSPDS3 * (LOC107780228)	CACATTGGTGATGGAGTTGC	GCAATTGGCAACGATTTCTT
*NtSPDS4* (LOC107765582)	GGAAAGGTGCTTGTTTTGGA	GAGACACCTCACGCAAGACA
*NtSPDS5* (LOC107810231)	AATTCCAAACCCCAAAAAGG	TGCAACTCCATCACCAACAT
*NtSPMS* (LOC107770315)	CGGTGTACTTTGCAACATGG	GGTCGAGCACAGAAGAAACC
*NtFT4 *(LOC107781882)	TGGTCGTGTGGTAGGAGATG	AGTTGCGAAGATCATCCCCT
*GhSAMDC*_*1*_ (NM_001326741.1)	GCAATGCTTCAAACCGGGTGAC	CCATCTCTCCGCAACGGTATCC
*Actin *(X69885)	CCTGAGGTCCTTTTCCAACCA	GGATTCCGGCAGCTTCCATT

### Construction of the expression vector and plant transformation

The cDNA of 1,064 bp mORF (main open reading frame) in *GhSAMDC*_*1*_ (GenBank accession number: JN020148) from upland cotton was amplified using pfu DNA polymerase (Transgene, Beijing, China) and constructed in the pGWB17 vector by Gateway Technology^45^. The specific primers for constructing the expression vector contained the forward primer CACCATGGAGCCTTCTCCTCGGT and the reverse primer CAAGATCGCTTCCGGAATG. The vector was confirmed by sequencing, introduced into *Agrobacterium tumefaciens* strain *EHA105* by electroporation and transformed into the tobacco (*Nicotiana tabacum* cv. NC89) by *Agrobacterium*-mediated transformation^46^. Kanamycin (50 mg/L) was used for antibiotic selection, and 4 transgenic lines were obtained. All transgenic and wild-type lines were sown in pots containing soil and cultivated in the phytotron under long-day conditions (16 h light/8 h dark). The seeds were harvested and cultivated in MS medium, including 50 mg/L kanamycin, and an approximately 3:1 segregation ratio was evaluated by kanamycin resistance. Homozygous plants were chosen for kanamycin resistance again.

### Detection of free polyamines in tobacco plants

Free polyamines were identified using a modified high-performance liquid chromatography (HPLC) system as described previously^47^. Leaves from different samples were ground in liquid nitrogen; 0.5 g of powder was dissolved in 1.5 mL of perchloric acid (5%, v/v) and incubated for 1.5 h at 4 °C. The mixture was centrifuged at 12,000 rpm for 20 min, and 150 μL of the supernatant was mixed with 200 μL of 2 M NaOH and 5 μL of benzoyl chloride, which was incubated at 37 °C for 30 min. Then, 500 μL of ether and 400 μL of saturated NaCl were added to the mixture, and the mixture was vortexed for 1 min at 25 °C. The mixture was centrifuged at 6,000 rpm for 10 min, and the organic phase was vacuum-evaporated. The mixture was vortexed for 5 min, and the polyamines were dissolved in 100 μL of methanol. Subsequently, the mixture was centrifuged at 6,000 rpm for 5 min and dissolved in 500 μL of methanol. HPLC was performed on an Agilent 1,200 system (Agilent Technologies, Palo Alto, CA, USA) with an Agilent XDB-C18 (4.6 mm × 150 mm) column. All experiments were run with three biological replicates each time.

### Statistical analysis

The SPSS ver. 16.0 statistical analysis package (SPSS Inc., Chicago, IL, USA) was used to perform the analysis of variance in this study. Differences between the average values were compared with Tukey’s HSD (Honestly significant difference) pairwise comparison test at the 5% confidence level.
